# Evaluation of Lapatinib Powder-Entrapped Biodegradable Polymeric Microstructures Fabricated by X-Ray Lithography for a Targeted and Sustained Drug Delivery System

**DOI:** 10.3390/ma8020519

**Published:** 2015-02-05

**Authors:** Eun-Goo Jeong, Hyung Jung Yoo, Byeonghwa Song, Hwang-Phill Kim, Sae-Won Han, Tae-You Kim, Dong-Il Dan Cho

**Affiliations:** 1 Cancer Research Institute, Seoul National University College of Medicine, Seoul 110-799, Korea; E-Mails: megablast@snu.ac.kr (E.-G.J.); phill1@snu.ac.kr (H.-P.K.); saewon1@snu.ac.kr (S.-W.H.); 2 Inter-university Semiconductor Research Center, Automation System Research Institute, School of Electrical Engineering and Computer Science, Seoul National University, Seoul 151-744, Korea; E-Mails: yhj@snu.ac.kr (H.J.Y.); bhsong@snu.ac.kr (B.S.); 3 Department of Internal Medicine, Seoul National University Hospital, Seoul 110-744, Korea; 4 Department of Molecular Medicine and Biopharmaceutical Sciences, Graduate School of Convergence Science and Technology, Seoul National University College of Medicine, Seoul 110-799, Korea

**Keywords:** drug delivery system (DDS), molecular targeted therapy, lapatinib, gastric cancer, sustained drug release, biodegradable polymer, polycaprolactone (PCL)

## Abstract

An oral medication of a molecular targeted drug, lapatinib, is taken regularly to maintain the drug concentration within the desired therapeutic levels. To alleviate the need for such cumbersome administration schedules in several drugs, advanced drug delivery systems (DDSs), which can provide time-controlled and sustained drug release, have recently received significant attention. A biodegradable synthetic polymer, such as polycaprolactone (PCL), is usually used as a carrier material for DDSs. In this paper, lapatinib powder-entrapped, PCL microstructures were fabricated with a precise X-ray lithography-based method. *In vitro* experiments on HER2 positive-human gastric cancer derived NCI-N87 cells were performed to appraise the drug release characteristics of the fabricated DDSs. The *in vitro* results indicate that after the X-ray lithography process, the lapatinib powder is still working well and show time- and dose- dependent drug release efficiencies. The cell growth inhibition characteristics of one hundred 40-μm sized microstructures were similar to those of a 1 μM lapatinib solution for over 144 h. In conclusion, the developed lapatinib-entrapped PCL microstructures can be used in molecular targeted delivery and sustained release as effective cancer-targeted DDSs.

## 1. Introduction

Chemotherapy represents a mainstay in the treatment of metastatic cancer. Although its efficacy has been demonstrated against most types of solid tumors, chemotherapy has two critical drawbacks: Toxicity and non-selectivity. Most anti-cancer drugs affect proliferating normal cells, such as digestive mucosa, hair follicles, and blood cells, resulting in various systemic toxicities. The delivery of chemotherapy is hampered by the presence of bio-barriers, such as the reticuloendothelial system, high interstitial pressure, and abnormal blood flow in tumors. To overcome these obstacles to chemotherapy, the development of molecular targeted therapy has been investigated. Molecular targeted agents are highly effective in cancers that carry characteristic genetic alterations [1,2]. For example, 25% of breast cancer cases present with activated HER2 signaling, and trastuzumab (known as Herceptin), a humanized monoclonal antibody for HER2, is highly effective in HER2-positive breast cancer [3,4]. Currently, more than 20 targeted agents are actively used in clinics [5]. These include gefitinib (known as Iressa) for EGFR mutant lung cancer [6,7] and imatinib (known as Gleevec) for c-kit positive gastrointestinal stromal tumors [8,9].

Lapatinib (known as GW572016, Tykerb) is a dual tyrosine kinase inhibitor (TKI) of epidermal growth factor receptor (EGFR/ErbB1) and human epidermal growth factor receptor 2 (HER2/ErbB2), which are potential drug targets [10,11,12,13]. Lapatinib was approved by the United States Food and Drug Administration (US FDA) in 2007 for use, in combination with capecitabine, in the treatment of patients with advanced or metastatic breast cancer whose tumors overexpress HER2 and who have received prior therapy, including anthracycline, taxane, and trastuzumab [14]. The HER2 gene, as the key target for lapatinib, is amplified in 15%–20% of breast cancers [15,16,17] and 5%–25% of gastric cancers [18,19,20,21,22]. HER2 overexpression cell lines, such as SNU-216, SK-BR-3 and NCI-N87 gastric cancer cell lines, are hypersensitive to lapatinib. In contrast, MET-amplified gastric cancer cell lines, such as SNU-638 and SNU-5, are resistant to lapatinib [23]. In our previous studies, we found the anti-tumor activity of lapatinib in gastric cancer with *in vitro* and *in vivo* molecular-based approaches, which can be used for targeted drug delivery [23,24,25].

Many orally administered drugs, including lapatinib, are not suitable for time-controlled and sustained drug release because they are intended to have systemic effects with a release profile of a half sine curve [26,27]. Therefore, patients are regularly administered doses to maintain the desired therapeutic levels of the drug throughout their medical treatments. For example, lapatinib in tablet form has effective plasma levels of 6 or 24 h, and the frequency of lapatinib dosing is one or three times daily [28]. To ameliorate the frequent dosing requirements in many drugs, drug delivery systems (DDSs) have recently received considerable attention, attributed to their high efficiency, low toxicity, and sustained drug release possibilities [29,30,31].

In the development of DDSs, there have been many possible candidates for drug carriers, such as cells, micelles, dendrimers, liposomes and biodegradable polymers. For a controlled and sustained DDS, however, the drug carrier should release the entrapped drug in a controlled manner. To ensure safety and maintain the desired therapeutic levels by time-controlled drug release, biodegradable polymers, such as polyethylene glycol (PEG), poly (lactic-co-glycolic acid) (PLGA), poly (lactic acid) (PLA), and polycaprolactone (PCL), have been used as DDS carriers. Among these polymers, PCL is most commonly selected for the following reasons: (1) its slow degradation typically taking two to three years to dissolve, which is suitable for controlled and sustained drug release; (2) its proven stability in serum; (3) its low material costs; and (4) the ability to use common microfabrication methods for DDSs [32,33]. One should note that various PCL polymers are approved by the US FDA for various human applications, such as scaffolds for tissue engineering, temporary prostheses, and drug delivery vehicles [34].

The drug release profile is affected by the shape of the drug carriers and how the drug is loaded. Lapatinib powder-entrapped microstructures can be microfabricated by UV lithography, laser cutting, microstamping, or X-ray lithography. However, the PCL polymer cannot be patterned with UV lithography. Laser cutting and microstamping are not suitable methods for precise shape patterning due to thermal damages and internal stress [33]. One should note that polymer deformation and reflow can plug the drug release openings. Therefore, X-ray lithography was used for precise shape patterning in this paper.

This paper developed a novel DDS that consists of PCL outer layers and a lapatinib-powder core for use in targeted and sustained drug delivery. The lapatinib powder-entrapped PCL microstructures 40 μm in size were fabricated with a lamination process and X-ray lithography process. The entrapped lapatinib powder in the PCL microstructure can be released only through the narrow side openings, and cannot be released through the outer-layer PCL films. A controlled and sustained release of drugs can be achieved continuously until the outer-layer PCL films completely dissolve away. Note that a slow-release paclitaxel loaded DDS intratumorally injected directly at tumor sites has shown the possibility of reducing the dosing frequency [35]. We are expecting similar results, and *in vitro* experiments with the developed lapatinib powder-entrapped DDS in this paper support this. The drug release characteristics of the fabricated DDS were evaluated with *in vitro* experiments in NCI-N87 cells.

## 2. Results and Discussion

### 2.1. Fabrication Results of Lapatinib Powder-Entrapped Microstructures

**Figure 1:**
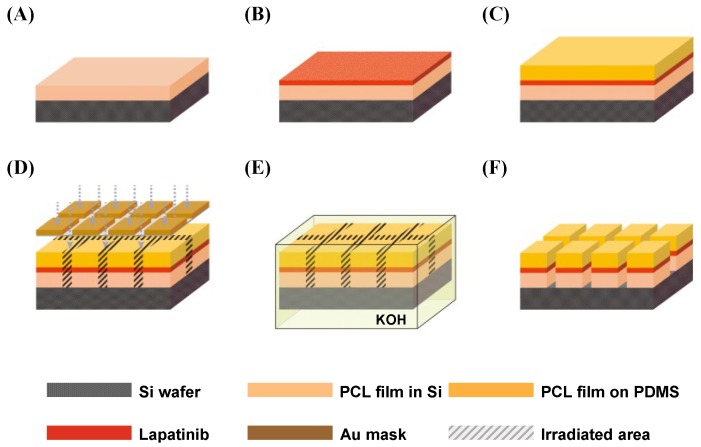
Illustration of X-ray lithography-based microfabrication methods. (**A**) A PCL film was spin-coated on a Si wafer; (**B**) Lapatinib powder was spread onto the PCL film; (**C**) Lapatinib powder was entrapped between two laminated PCL films with a lamination process; (**D**) Lapatinib powder-entrapped PCL films were exposed to X-ray synchrotron radiation through the X-ray mask; (**E**) X-ray irradiated areas were etched out in KOH during the development process; (**F**) Fabricated lapatinib powder-entrapped microstructures.

The fabrication process is shown in Figure 1. The polymeric solution was first prepared by dissolving PCL in dichloromethane (DCM) at 10% (*w*/*v*). The solution was poured onto a four-inch silicon wafer and a poly(dimethylsiloxane) (PDMS) film and spin-coated at 1400 revolutions per minute (RPM) for 35 s to formulate PCL films of 10 µm thickness shown in Figure 1A. The lapatinib powder was spread onto the PCL film shown in Figure 1B. Then, the lapatinib powder spread PCL film was covered with a second PCL film formulated on a PDMS substrate. The wafer was heated at 65 °C on a hot plate and compressed with a roller for 5 min. Thus, the lapatinib powder was entrapped between the two layers of PCL films through the lamination process. Then, the PDMS coated wafer was detached, and lapatinib powder-entrapped PCL films were fabricated as shown in Figure 1C.

The silicon wafer with the lapatinib powder-entrapped PCL films was exposed to X-ray synchrotron irradiation through a mask with square-shaped patterns shown in Figure 1D. The irradiated areas were etched out in a potassium hydroxide (KOH) solution shown in Figure 1E. Finally, non-irradiated PCL films were rinsed with deionized water, which completed the fabrication of the lapatinib powder-entrapped microstructures shown in Figure 1F.

The X-ray synchrotron irradiation changes the polymer properties by breaking the main chain of PCL. The main chain scission in the PCL film causes an increase in the hydrolysis rate when the PCL film is immersed in an alkaline solution [36]. As a consequence, the X-ray synchrotron-irradiated areas of the laminated PCL film are further hydrolyzed than the non-irradiated areas of the laminated PCL film in a 45% (*w*/*v*) KOH solution. During hydrolysis in the KOH solution, the irradiated lines are separated from the non-irradiated areas. Then, the non-irradiated areas become the final microstructures with entrapped lapatinib.

To avoid chemical damages (degradation or discoloration) to lapatinib during the X-ray lithography process, the exposure to the X-ray synchrotron irradiation must be below a certain threshold level. Varshney and colleagues have reported that cyclophosphamide (CPH) and doxorubicin hydrochloride (DOXO) do not undergo degradation or discoloration after being irradiated by gamma radiation of 5000 Gy, which is equivalent to approximately 7 J/cm^3^ of X-ray synchrotron irradiation [37]. In this experiment, we exposed the laminated lapatinib and PCL films for 45 min with a beam current of 200 mA. Under this condition, the irradiation dose to entrapped lapatinib is approximately 2 J/cm^3^, which is well below the threshold level.

**Figure 2:**
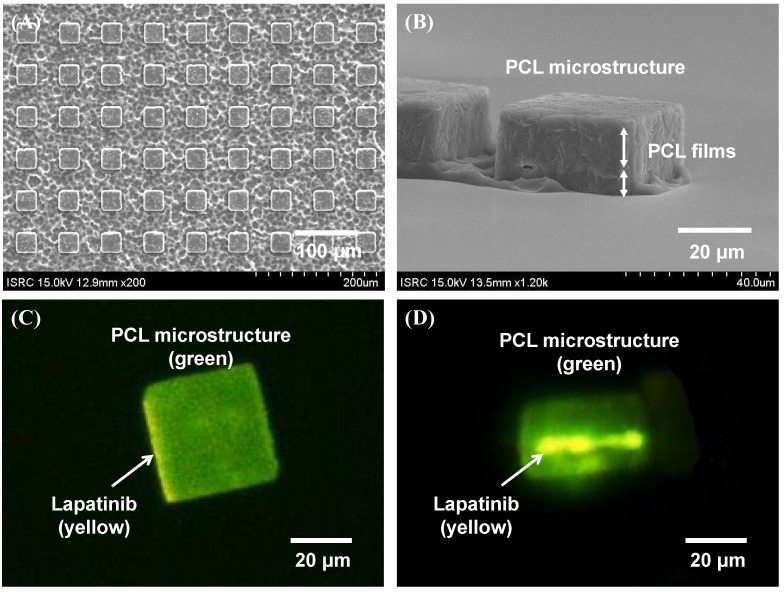
Images of fabricated lapatinib powder-entrapped PCL microstructures. (**A**) Top view of the microstructures by SEM during the development process; (**B**) Side view of the microstructures by SEM after the development process; (**C**) Top view of a microstructure by fluorescent microscope; (**D**) Side view of a microstructure by fluorescent microscope.

The images of the fabricated lapatinib powder-entrapped microstructures are shown in Figure 2. Figure 2A shows the different hydrolysis rate of the PCL films during the development process. The measured length and height of the microstructure are 40 µm and 20 µm, respectively, shown in Figure 2B. In the scanning electron microscope (SEM) images in Figure 2A,B, the lapatinib powder is not apparent, but in the fluorescent microscope images in Figure 2C,D, the lapatinib powder is clearly visible. The side-view fluorescent microscope image in Figure 2D shows that the lapatinib powder was successfully entrapped in the middle of the PCL films. That is, the image shows that the microstructure is green, which is the green fluorescence in the PCL material. The color of lapatinib is yellow, which is visible only on the sides (more apparent on the left side due to a slight tilt) of the microstructure in Figure 2C and is visible between the PCL films in Figure 2D. Note that in Figure 2A, these lapatinib-entrapped microstructures were batch processed, and more than 10 million 40 × 40 μm^2^ microstructures can be fabricated in a one 8-inch wafer in a single-mask process step.

### 2.2. Evaluation of Lapatinib Powder-Entrapped Microstructure Activity

#### 2.2.1. *In Vitro* Drug Release Characteristics of the Lapatinib Powder-Entrapped Microstructures

**Figure 3:**
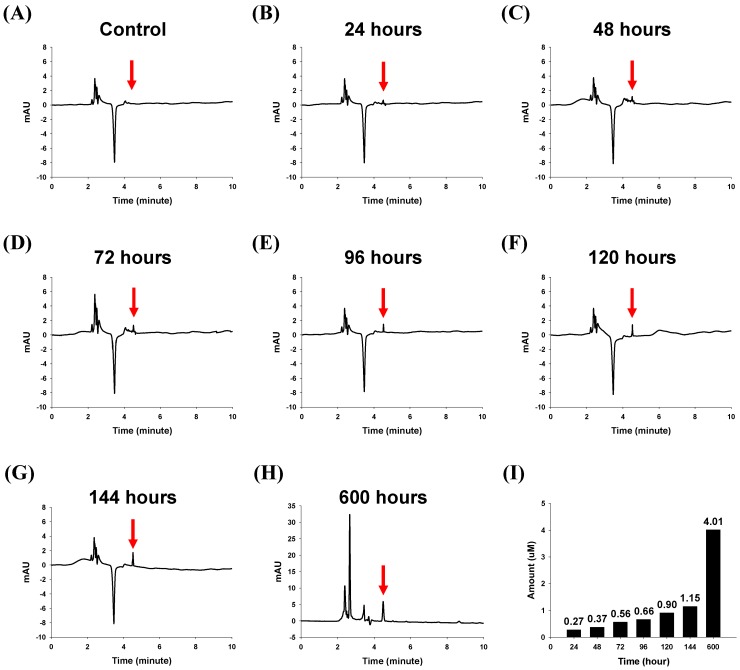
HPLC analysis results from one hundred of the 40-μm sizedmicrostructures in PBS solution. (**A**) Chromatogram of the microstructures without lapatinib powder; (**B)**–(**H**) Chromatograms of the lapatinib powder-entrapped microstructures after 24, 48, 72, 96, 120, 144, and 600 h; (**I**) Amounts of lapatinib released from one hundred of the 40-μm sized microstructures after 24, 48, 72, 96, 120, 144, and 600 h were 0.27, 0.37, 0.56, 0.66, 0.90, 1.15, and 4.01 μM, respectively.

The *in vitro* drug release characteristics of the lapatinib powder-entrapped microstructures were investigated. To validate the amount of lapatinib released from the fabricated lapatinib powder-entrapped microstructures, high performance liquid chromatography (HPLC) was used. The microstructures without lapatinib powder in a phosphate buffered saline (PBS) solution had no peak at the retention time of 4.49 min, as shown in Figure 3A. One hundred 40-μm sized, lapatinib powder-entrapped microstructures in the PBS solution after 24, 48, 72, 96, 120, 144, and 600 h were analyzed. Each case had a sharp peak at the retention time of 4.49 min, and the peaks had different heights, as shown in Figure 3B–H. The amounts of released lapatinib corresponding to the samples after 24, 48, 72, 96, 120, 144, and 600 h were measured as 0.27, 0.37, 0.56, 0.66, 0.90, 1.15, and 4.01 μM, respectively, as shown in Figure 3I. The percentages of released lapatinib corresponding to the samples after 24, 48, 72, 96, 120, 144, and 600 h were then calculated as 1.59%, 2.18%, 3.30%, 3.88%, 5.31%, 6.75%, and 23.60%, respectively. The results show the time-dependent release characteristics of the fabricated lapatinib powder-entrapped microstructures.

#### 2.2.2. Growth Inhibitory Activity in HER2-Amplified Gastric Cancer Cell Lines by Lapatinib after the Lamination Process

**Figure 4:**
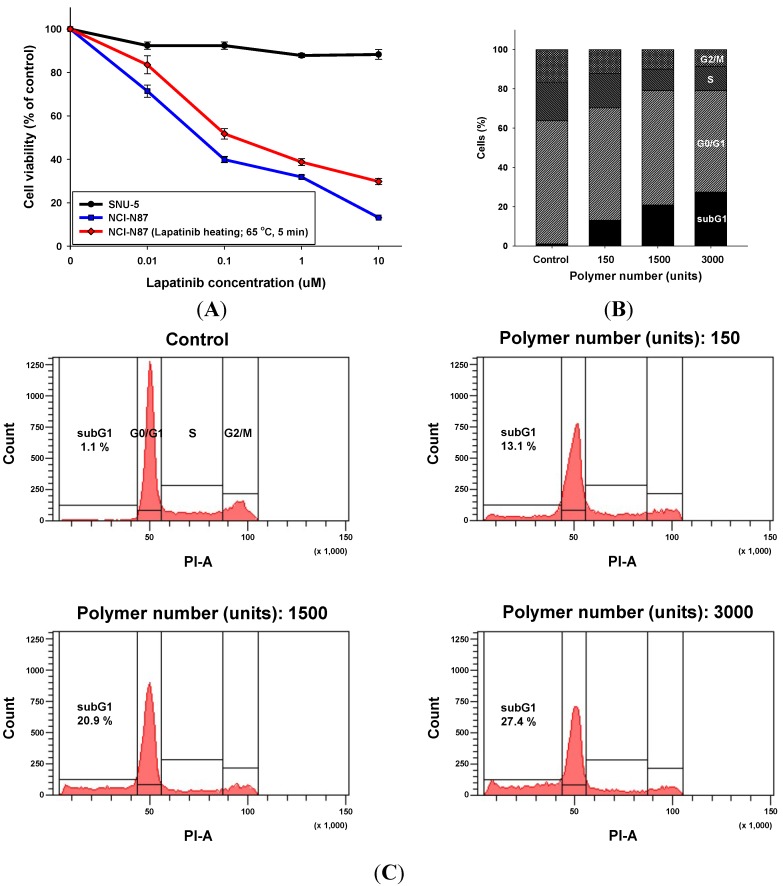
Effects of lapatinib and lapatinib powder-entrapped microstructures on gastric cancer cell lines. (**A**) Cells were plated at 3000 cells/well in a 96-well plate. After 24 h, cells were treated with lapatinib at various concentrations (0.01–10 μM). After 72 h, an MTT assay was performed to evaluate the growth inhibitory activity. The absorbance of each well was measured at 540 nm. The percentage of growth is expressed as a percentage of the control (no drug) cell growth; (**B**) NCI-N87 was treated with lapatinib-entrapped microstructures (150, 1500 and 3000 units per 3 mL of RPMI-1640 medium) for 72 h. After the treatment schedule, cells were analyzed for DNA content by flow cytometry. Proportions of the cells in the subG1, G0/G1, S and G2/M phases were quantified with the FACS Diva program; Control (subG1: 1.1%, G0/G1: 62.6%, S: 19.5%, G2/M: 16.8%), 150 units (subG1: 13.1%, G0/G1: 57.2%, S: 17.5%, G2/M: 12.2%), 1500 units (subG1: 20.9%, G0/G1: 58.3%, S: 10.8%, G2/M: 10.0%) and 3000 units (subG1: 27.4%, G0/G1: 51.8%, S: 12.2%, G2/M: 8.6%); (**C**) Cell cycle phases displayed as a histogram.

HER2 amplification is a significant predictive marker for the growth inhibitory activity of lapatinib in breast and gastric cancers. To validate the growth inhibitory activity of lapatinib, NCI-N87 (a HER2-amplified gastric cancer cell line) and SNU-5 (a MET-amplified gastric cancer cell line, as a control) were used. Both cell lines were treated with lapatinib solutions at 0.01, 0.1, 1 and 10 μM for three days, shown in Figure 4A. Because the wafers were heated at 65 °C on a hot plate and compressed by a roller for 5 min during the lamination process, we simulated the damage to drug efficiency by heating the lapatinib stock solutions at the same condition (65 °C for 5 min) using a heat block and evaluated the cell inhibitory ability for over 72 h by MTT (3-[4,5-dimethylthiazol-2-yl]-2,5-diphenyltetrazolium bromide) assay. As shown in Figure 4A, the IC_50_ levels in the NCI-N87 cells for the lapatinib solutions and heated lapatinib were 0.052 and 0.1 μM, respectively, which was similar to a value in a previous report [23]. The activity of the heated lapatinib solutions was somewhat decreased, and the growth inhibitory activity showed a concentration-dependence in HER2 positive NCI-N87 cell lines shown in Figure 4A. Therefore, the high temperature during the fabrication process has a small effect on the growth inhibitory activity of lapatinib.

#### 2.2.3. Apoptotic Cell Death by the Lapatinib Powder-Entrapped Microstructures

According to our previous studies, lapatinib-inhibited phosphorylation of HER2, EGFR and downstream signaling proteins resulting in G1 arrest and induction of apoptosis [23]. To assess induced cell death and the apoptotic ability of the lapatinib powder-entrapped microstructures, we performed cell cycle analysis in the NCI-N87 cell lines. The cell cycle was analyzed with a FACS-Canto II flow cytometer. The NCI-N87 cells were treated with the lapatinib powder-entrapped microstructures at different concentrations (150, 1500 and 3000 units per 3 mL of RPMI-1640 medium) for over 72 h. Considering the relative sensitivity of the lapatinib solutions, the NCI-N87 cells were treated under the same conditions.

As a result, in a concentration-dependent manner, the subG1 phase fraction was significantly increased, as shown in Figure 4B,C. The subG1 phase fractions of the lapatinib-entrapped microstructures were calculated as 13.1%, 20.9% and 27.4% compared to 1.1% for the control when the number of the microstructures was 150, 1500 and 3000 units, respectively. Moreover, G2/M phase cell cycle block was also decreased at 12.2% for 150 units, 10.8% for 1500 units and 8.6% for 3000 units compared to 16.8% for the control. Therefore, lapatinib powder-entrapped microstructures may induce apoptotic cell death. Conversely, our lapatinib powder-entrapped biodegradable polymeric microstructures fabricated by X-ray lithography worked well in the *in vitro* systems.

### 2.3. Drug Release Efficiency of the Lapatinib Powder-Entrapped Microstructures

#### 2.3.1. Size- and Dose-Related Drug Release Efficiency Test

**Figure 5:**
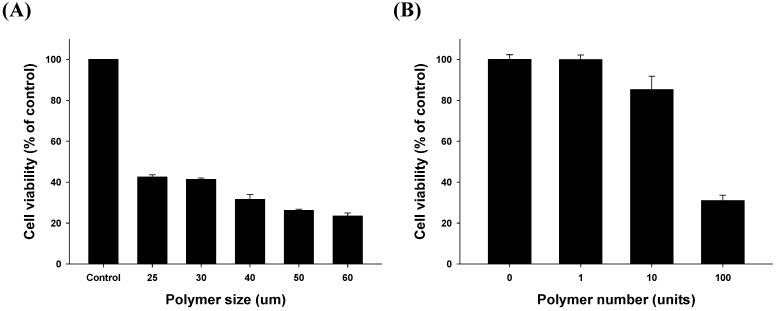
Evaluation of size- and dose-related drug release efficiency (**A**) Cell growth inhibitory activity of various sized (25, 30, 40, 50 and 60 μm) microstructures was evaluated with MTT assays. Each test group was treated with the same number of units (100 units) and same treatment time (72 h); (**B**) Fixed size (40 μm) and varying numbers of units (1, 10 and 100 units) tested over 72 h.

We hypothesized that the size and number of lapatinib powder-entrapped microstructures influence drug release and result in different efficiencies of lapatinib-induced cell death. First, we tested the size-dependent efficiency. Our microstructures were fabricated at various sizes including 25, 30, 40, 50, and 60 μm. As shown in Figure 5A, for the same number of microstructures, cell growth was further suppressed as the size of the fabricated lapatinib powder-entrapped microstructures increased. Cell viabilities were calculated as 42.5%, 41.4%, 31.6%, 26.2%, and 23.4% compared to the control group when the size of the microstructures was 25, 30, 40, 50, and 60 μm, respectively. These results suggest that the entrapped lapatinib powder in the laminated PCL films cannot be released through the outer laminated PCL films, which allows drug release only through the unblocked area. The amount of drug released from the fabricated lapatinib powder-entrapped microstructures increases as the unblocked area of lapatinib increases.

Second, we also evaluated the dependence on the number of units administered. In the case of the 40-μm sized microstructures, cell growth was further suppressed as the number of the lapatinib powder-entrapped microstructures increased shown in Figure 5B. Cell viabilities were calculated as 99.8%, 85.2%, and 30.9% compared to the control group when the unit of the microstructures varied from 1, 10, and 100, respectively. These results suggest that as the number of the microstructures increases, the amount of lapatinib released from the microstructures also increases. Therefore, the controlled release of drugs can be easily achieved by controlling the number of lapatinib powder-entrapped microstructures.

#### 2.3.2. Time-Related Drug Release Efficiency Test

**Figure 6:**
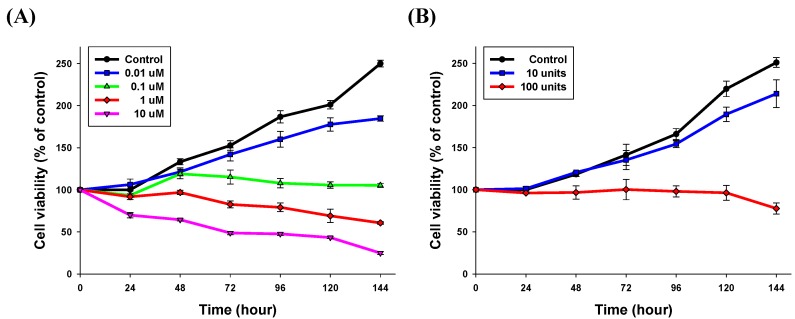
Evaluation of time-related drug release efficiency. (**A**) As a control, lapatinib solution was used in the MTT assay for 24 to 144 h; (**B**) With the 40 μm microstructures, the cell growth inhibitory efficiency was measured with varying numbers of units (10 and 100 units) until 144 h.

We assessed the time-dependent manner of lapatinib powder-entrapped microstructure delivery to examine the potential of sustained drug delivery. As shown in Figure 6, cell growth was successfully suppressed for over 144 h. With the 40-μm sized microstructure and 100 units, the cell viabilities compared to the control group in the initial day were calculated as 100.6%, 104.2%, 101.9%, 100.2%, and 80.8%, respectively. Compared to the case of NCI-N87 cells solely treated with 1 μM of lapatinib solution, the 40-μm sized lapatinib powder-entrapped microstructures were similar in the presented conditions. For one hundred of the 40-μm sized microstructures, the lapatinib powder was entrapped with a concentration of 17 μM. Therefore, the fabricated lapatinib powder-entrapped microstructures released 6% of the entrapped lapatinib over 144 h, and still can release the remaining lapatinib powder over a longer period.

### 2.4. Applications of Lapatinib Powder-Entrapped Microstructures

The final purpose of DDSs is to achieve a suitable bioavailability for the target areas of the human body. To enhance the bioavailability of drugs, two characteristics are required: (1) long-term release of drugs within the desired therapeutic levels and (2) targeted delivery of drugs to specific areas. The PCL polymer has a very slow degradation rate of two to three years; therefore, it is an appropriate carrier material for the long-term release of drugs. The release rate of drugs can also be achieved by varying the number of microstructures and/or by changing the size of the release channel for the microstructures.

Our novel DDS idea can be applied to various uses in oncology. First, our microstructures can be applied to other major molecular target agents in several different cancers. There are more FDA approved molecular target agents, other than lapatinib, that we selected. In our further studies, we will entrap various molecular target agents, including gefitinib (for EGFR mutant lung cancer) and imatinib (c-kit positive gastrointestinal stromal tumors). In addition, our system may be applicable in targeted specific gene knockdown by entrapped si-RNAs or microRNAs. In practice, RNA interference (RNAi) is a powerful and successful approach in loss of function studies. We expect that these small RNAs also can be applied to our entrapped-system idea.

## 3. Experimental Section

### 3.1. Biodegradable Polymers and Molecular Targeted Anti-Cancer Drug for the DDS

As a carrier material of DDSs, PCL was selected in this paper because of its proven biodegradability and safety. PCL was purchased from Sigma-Aldrich Korea Ltd. (Yongin, Gyeonggi-do, Korea) and stored in a dry and well-ventilated environment.

Lapatinib was selected because of its hypersensitivity to HER2 positive cancer cell lines and its yellow color, which can be easily visualized. Lapatinib was provided by GlaxoSmithKline (Research Triangle Park, NC, USA). Stock solutions of this chemical were prepared in dimethyl sulfoxide and stored at −20 °C.

### 3.2. X-ray Lithography-Based Microfabrication Method

The X-ray lithography process was performed at the Pohang Accelerator Laboratory (PAL) [38]. The silicon wafer with the lapatinib-entrapped PCL films and an X-ray mask was placed together onto a jig, and then, X-ray synchrotron radiation was exposed throughout the jig by controlling the scanning servo motor system. An X-ray mask made of gold (Au) with square-shaped patterns was fabricated with a conventional micro-electromechanical system (MEMS). A scanning electron microscope (SEM), a BHMJL optical microscope (Olympus, Tokyo, Japan) and an IX51 inverted fluorescent microscope (Olympus) were used to observe the fabricated lapatinib powder-entrapped microstructures.

### 3.3. High Performance Liquid Chromatography for the in Vitro Drug Release Test

For the *in vitro* drug release test, one hundred 40-μm sized lapatinib powder-entrapped microstructures in PBS solution after 24, 48, 72, 96, 120, 144, and 600 h were analyzed using the Ultimate 3000 (Thermo Dionex, Sunnyvale, CA, USA) HPLC system and Acclaim C_18_ column (250 × 4.6 mm, 5 μ, Thermo, Waltham, MA, USA). Potassium dihydrogen phosphate (KH_2_PO_4_, 0.02 M, pH 2.8) and methanol (MeOH) in a ratio of 80:20 (*v*/*v*) were selected as a suitable mobile phase for the estimation of lapatinib. All solvents were HPLC grade, and the prepared sample solutions and the mobile phase were stored at room temperature. Ten microliters of the lapatinib sample solutions were analyzed after filtering with 0.45 μm membrane filter. The lapatinib powder in the PBS solution at 0.5 μM was used to find the standard chromatogram of lapatinib. To find the desired wavelength, the photodiode array detector was used in a scan mode with a scan range of 200–800 nm and the desired peak coverage of 100%. The flow rate of the mobile phase was maintained at 1 mL/min. The oven temperature was 30 °C. A sharp peak at the retention time of 4.49 min was observed in a wavelength of 220 nm with a run time of 10 min. A calibration curve was determined by taking the absorbance values corresponding to the lapatinb solutions at 0.5, 5 and 25 μM. The percentage of released lapatinib from the DDS was calculated using the formula:

$$\text{Released lapatinid (\%)}=\frac{\text{Amount of released lapatinib}}{\text{Amount of entrapped lapatinib in the microstructures}}\times 100.$$

### 3.4. Cell Culture

The mycoplasma free human gastric cancer cell lines, NCI-N87 and SNU-5, were obtained from the Korean Cell Line Bank (Seoul, Korea) [39,40]. Both cell lines were cultured in RPMI-1640 complete medium (WelGENE, Daegu, Korea) with 10% fetal bovine serum (WelGENE, Daegu, Korea) and 1% gentamicin (Cellgro, Manassas, VA, USA) at 37 °C under 5% CO_2_ and humidified atmosphere.

### 3.5. Cell Cycle Analysis

Cells were washed twice in phosphate-buffered saline solution, fixed in 70% ethanol and incubated at −20 °C until the analysis was performed. Before the analysis, cell suspensions were washed with phosphate-buffered saline solution, digested with RNase A (50 μg/mL) for 15 min at 37 °C and stained with propidium iodide (50 μg/mL) as previously described [23]. The DNA content (10,000 cells/experimental group) was determined with a FACS-Canto II flow cytometer (BD Biosciences, San Jose, CA, USA) equipped with the FACS Diva program (BD Biosciences). Cell-cycle populations were analyzed with SigmaPlot, v.10.0 (Systat, San Jose, CA, USA).

### 3.6. Growth Inhibition Assays

The viability of cells was assessed with the MTT assay (Sigma-Aldrich, St. Louis, MO, USA). A total of 3 × 10^3^ cells were seeded in 96-well plates, incubated for 24 h, and treated from 24 to 144 h at 37 °C with the indicated drugs. Following treatment, MTT solution was added to each well and incubated for 4 h at 37 °C. The medium was then removed, and dimethyl sulfoxide was added and mixed thoroughly for 30 min at room temperature. Cell viability was determined by measuring the absorbance at 540 nm with a VersaMax Microplate Reader (Molecular Devices, Sunnyvale, CA, USA), and data were analyzed with the SoftMax Pro Software (Molecular Devices) comprehensive data analysis software. The concentration of the drug required to inhibit cell growth by 50% was determined via interpolation from dose-response curves with SigmaPlot, v.10.0 (Systat). Six replicate wells are presented as the mean number of the remaining cells with 95% confidence intervals.

### 3.7. Statistics

All experiments were conducted in duplicate or triplicate, with at least two biological replicates. All data are expressed as the mean ± SD (standard deviation). Statistical significance was calculated with an unpaired Student’s *t*-test, and values of *p* < 0.05 were considered statistically significant.

## 4. Conclusions

For targeted and sustained drug delivery, a novel DDS that maintains the drug concentration within the desired therapeutic levels was presented in this paper. Lapatinib powder and PCL were selected to fabricate drug-entrapped microstructures using an X-ray lithography-based microfabrication method. The fabrication method produced well-patterned microstructures, and the method is scalable for mass production. The sustained drug release characteristics were evaluated by *in vitro* experiments in NCI-N87 cells. The *in vitro* results showed that lapatinib has a slightly decreased effectiveness after the lamination and X-ray synchrotron irradiation processes; however, the processing effects are minimal. The microfabricated DDS showed sufficiently effective activities in controlling the cell growths of HER2-amplified gastric cancer for over 144 h. Our final goal is to extend the developed DDS method to clinical applications for targeted and sustained drug delivery, and as our next step, *in vivo* experiments with mice are currently under development.
